# Macrophage Transcriptomic Alterations Driven by Alphavirus-Based Cancer Immunotherapy Vectors

**DOI:** 10.1155/jimr/6573891

**Published:** 2025-06-13

**Authors:** Ksenija Korotkaja, Darija Lapina, Zhanna Rudevica, Anna Zajakina

**Affiliations:** Cancer Gene Therapy Group, Latvian Biomedical Research and Study Centre, Ratsupites Str. 1 k. 1, Riga LV-1067, Latvia

**Keywords:** alphavirus, bone marrow-derived macrophages, cytokine gene delivery, Semliki Forest virus, tumour microenvironment

## Abstract

Cancer cells promote the polarisation of tumour-associated macrophages (TAMs) into pro-tumorigenic M2-like phenotype, contributing to cancer progression. Reprogramming TAMs by viral immunotherapy vectors represents a promising strategy for cancer therapy. However, the factors driving macrophage reprogramming into a tumour-suppressing M1-like phenotype in response to viral vectors remain unclear. Alphaviral vectors, such as Semliki Forest virus (SFV), indirectly influence macrophages through cancer cell infection, cytokine gene delivery and tumour microenvironment (TME) modulation. This study examines macrophage transcriptomic alterations induced by SFV vectors. Murine mammary cancer cells were infected with SFV delivering tumour necrosis factor-α (TNFα) or interferon-γ (IFNγ) genes. Conditioned media from infected cells were used to treat bone marrow-derived macrophages (BMDMs) with subsequent analysis of the transcriptome. As a result, SFV-infected cancer cells significantly altered cytokine and chemokine profiles, reducing immunosuppressive factors (e.g., IL-10) and increasing inflammatory mediators (e.g., CXCL10 and CCL4). RNA sequencing revealed upregulation of genes associated with antigen presentation, interferon responses and M1 polarisation in macrophages treated with SFV/TNFα and SFV/IFNγ-conditioned media. SFV/IFNγ inhibited cancer-associated pathways (angiogenesis, glycolysis and extracellular matrix (ECM) remodelling) and enhanced cytotoxic lymphocyte (CTL) chemoattractants (CXCL9 and CXCL10). SFV/TNFα selectively upregulated *Mmp2*, *Mmp14* and *Ccl22*. All SFV vectors upregulated PD-L1 (*Cd274*) expression. The study demonstrates that alphavirus-mediated gene delivery to cancer cells can impact macrophages, inducing proinflammatory responses and reprogramming them into anti-cancer phenotype. However, combining SFV/IFNγ with immune checkpoint inhibitors could potentially improve therapeutic efficacy by mitigating virus-induced suppressive signals in the TME.

## 1. Introduction

Tumour-associated macrophages (TAMs) are the main component of the tumour microenvironment (TME), comprising up to 50% of the immune cell infiltrate in breast cancer [1]. The M1 phenotype supports anti-tumour immune responses, while the M2 phenotype promotes angiogenesis, metastasis and immune suppression [2]. Co-culture studies show that cancer cell-derived factors frequently induce TAMs to acquire an M2-like immunosuppressive phenotype [3, 4]. Moreover, cancer cell-derived factors activate macrophage migration and chemotaxis pathways [5].

Reprogramming macrophages towards the M1 phenotype is a promising strategy for cancer therapy. Viruses are highly effective candidates for delivering therapeutic genes due to their ability to selectively target cells and efficiently mediate transgene expression [6]. By harnessing their natural capacity for gene delivery, viral vectors enable precise genetic reprogramming. Alphaviruses, such as Semliki Forest virus (SFV) and Sindbis virus, are effective vectors for delivering therapeutic genes, possessing minimal pre-existing immunity and efficient transgene expression [6, 7]. SFV upregulates immunogenic cancer cell death, activating dendritic cells to produce Th1 cytokines and effectively cross-present antigens, leading to the activation of antigen-specific cytotoxic lymphocytes (CTLs) [8].

Compared to many other vectors that infect macrophages and deplete them [9], SFV vectors do not directly infect bone marrow-derived or blood-derived macrophages [10]. This feature represents an advantage of the vectors able to kill cancer cells and influence macrophage behaviour, activating their anti-tumour potential. Furthermore, SFV1 is a replication-deficient vector, which enhances its safety profile by preventing unintended viral replication and spread [7]. SFV1 replication-deficient particles are produced by co-electroporation of in vitro transcribed RNA from an expression vector and helper constructs, carrying virus structural genes. Although SFV therapy holds great potential, the precise effects of the recombinant virus on the TME, including immune cell dynamics and overall tumour progression, remain unexplored.

Oncolytic viruses (OVs) are known to promote the pro-inflammatory macrophage phenotype [11]. On the other hand, PD-L1 expression in tumours has been shown to increase following OV treatment, leading to the suppression of T-cell cytotoxicity [12]. Furthermore, numerous viruses such as hepatitis C virus (HCV), human papillomavirus (HPV), hepatitis B virus (HBV) and severe acute respiratory syndrome coronavirus 2 (SARS-CoV-2) are known to programme macrophages towards the M2 phenotype by increasing the expression of cellular inhibitory receptors, suppressing antigen presentation and decreasing proinflammatory cytokine production [13–15]. Moreover, virus strain pathogenicity can affect macrophage phenotype and modulate macrophage plasticity, as shown for arenaviruses [16].

Delivered cytokine genes may directly affect macrophage programming, which, together with virus-upregulated factors, can generate a synergistic effect. We have chosen two promising SFV vectors engineered to deliver tumour necrosis factor-α (TNFα) or interferon-γ (IFNγ), SFV1/IFNγ and SFV1/TNFα [10, 17]. IFNγ and TNFα have been previously explored in clinical trials for cancer therapy due to their potent immune-stimulatory effects [18]. However, their clinical application has been limited by significant toxicity and the activation of counter-regulatory mechanisms within the TME. SFV-mediated delivery can localise therapy to the tumour site, potentially avoiding the systemic toxicity and negative side effects associated with conventional treatments. By restricting therapeutic gene expression to the TME, replication-deficient SFV vectors offer a more targeted and safer approach to cancer therapy. This study evaluated the impact of SFV and SFV-mediated immunostimulatory gene delivery on macrophage programming, focusing on the anti-tumour phenotype and mitigating the pro-tumorigenic effects of cancer cell-derived factors on macrophages.

## 2. Methods

### 2.1. Cells

Murine mammary gland adenocarcinoma 4T1 cells (ATCC CRL-2539) were cultured in DMEM-GlutaMAX (Cat. No. 31966-021; Gibco), 10% fetal bovine serum (FBS; Cat. No. FBS-HI-12A; Capricorn Scientific) and 40 µg/mL gentamicin (Cat. No. 00-0442; Sopharma). Fibroblast-like L929 cells (ATCC CCL-1) were cultured in RPMI-1640 medium (Cat. No. 12-115F; Lonza BioWhittaker) supplemented with 10% FBS, 1% penicillin/streptomycin (PEST; Cat. No. 15070-063; Gibco) and 2 mM L-glutamine (Cat. No. 25030-024; Gibco). Baby hamster kidney fibroblasts (BHK-21, ATCC CCL-10) were cultured in BHK-Glasgow MEM (Cat. No. 21710-025; Gibco) supplemented with 5% FBS, 10% tryptose phosphate broth (Cat. No. 18050-039; Gibco), 2 mM L-glutamine, 20 mM HEPES (Cat. No. 15630-056; Gibco) and 1% PEST as described previously.

Mouse macrophages were obtained from bone marrow cells according to an established protocol using an L929 cell-conditioned medium (LCCM) as a source of macrophage colony-stimulating factor (M-CSF) [19]. Briefly, L929 cells were cultivated in a monolayer for 7 days; then, the LCCM was collected, clarified by centrifugation and frozen at −20°C. Bone marrow from the femurs and tibia of the hind legs of 8–12 weeks female BALB/c mice were used to generate bone marrow-derived macrophages (BMDMs) as described previously [17, 20]. BMDMs were cultivated in RPMI-1640 medium containing 10% FBS, 1% PEST, 2 mM L-glutamine and 30% LCCM. All animal experimental protocols were approved by the Latvian Animal Protection Ethical Committee of Food and Veterinary Service (Permit Nr. 93, from 11 December 2017, Riga, Latvia).

### 2.2. Production of Replication-Deficient Viral Particles

The pSFV1-TNFα and pSFV1-IFNγ vectors, encoding murine TNFα and murine IFNγ genes, respectively, were generated in our lab as described previously [10]. The pSFV1/Enh-Luc plasmid containing the firefly luciferase (*Luc*) gene was generously provided by A. Merits (Institute of Technology, University of Tartu, Estonia). Liljeström and Garoff [21] (Karolinska Institute, Sweden) have generously provided the pSFV1 and pSFV-Helper1 plasmids.

Replication-deficient viral particles were produced as described previously [10, 17, 22, 23]. To package the RNAs into viral particles, the recombinant RNA was co-electroporated with the SFV-Helper1 RNA in BHK-21 cells. The cell conditioned medium (CM) containing the infectious viral particles was purified and concentrated by ultracentrifugation as previously described [24]. The concentrated viral particles were frozen in liquid nitrogen and stored at −70°C. The infectious virus particle titres were determined by immunostaining and expressed as infectious units per mL (iu/mL).

### 2.3. Infection

Cells were cultivated to 80%–90% monolayer density. Cells were washed twice with PBS Ca^2+^/Mg^2+^, and then, the viral particle solution diluted with PBS Ca^2+^/Mg^2+^ was added to the cells. The cells were incubated at 37°C for 1 h. After incubation, the viral particle-containing solution was removed from each well and the medium supplemented with a reduced amount of FBS was added (5% for 4T1 cells, 1% for BHK-21 cells). Infected cells were incubated overnight in a humidified 5% CO_2_ incubator at 37 °C.

### 2.4. Experimental Design

4T1 cells (T-75) were infected with 8 × 10^7^ iu in 5.5 mL PBS Ca^2+^/Mg^2+^. Incubated for 1 h 10 min at 37°C, the virus was removed and 11 mL of 4T1 medium with 5% FBS was added to the cells. The cells were incubated for 48 h, after which the medium was collected and centrifuged for 5 min at 400 rcf. The medium was transferred to a new tube and centrifuged once more for 10 min at 5400 rcf. Following this, 0.5 mL of the medium was added to the BMDMs cultured in 2 mL RPMI-1640 medium containing 10% FBS, 1% PEST, 2 mM L-glutamine and 10% LCCM in a 6-well plate. After 24 h, RNA was extracted with RNeasy Mini kit (Cat. No. 74104; QIAGEN), treated with DNase I on the column (Cat. No. 79254; QIAGEN), eluted with 30 µL H_2_O and stored at −70°C. The RNA concentration was measured with a Qubit HS RNA kit (Cat. No. Q32855; Invitrogen) and quality was assessed with a BioAnalyzer RNA 6000 pico kit (Cat. No. 5067-1513; Agilent).

### 2.5. Analysis of Cytokines and Chemokines by ELISA and Luminex Assays

The cytokines and chemokines were quantified using Mouse IFNγ Uncoated ELISA (Cat. No. 887314; Invitrogen) and Mouse Chemokine Panel 31-Plex (Cat. No. 12009159; Bio-Rad, Hercules, CA, USA) according to the manufacturer's instructions. Visualisation and statistical analysis were performed using the GraphPad Prism 8.02 software. One-way ANOVA was employed to determine significant differences between treatment groups.

### 2.6. mRNA Sequencing

rRNA was depleted with MGIEasy rRNA Depletion Kit (Cat. No. 1000005953; MGI). The library was prepared with MGIEasy RNA Directional Library Prep Set (Cat. No. 1000006385; MGI). The quality and concentration of libraries were tested using Agilent 2100 BioAnalyzer and Qubit 2.0. We sequenced the pituisphere paired-end libraries on DNBSEQ-G400 sequencer (MGI, PRC) with 150 bp read length.

Raw sequencing data were assessed using FastQC (v0.12.1) [25] and summarised with MultiQC (v1.23) [26]. We trimmed the reads using fastp (v0.23.4) [27, 28], specifying adapter sequences for both forward and reverse reads. Trimming was performed to remove low-quality bases from the front and tail of the reads and to ensure an average Phred quality score of at least 20. Reads shorter than 75 base pairs were discarded. Trimmed reads were aligned to the *Mus musculus* BALB/cJ genome (Mus_musculus_balbcj.BALB_cJ_v1) using HISAT2 (v2.2.1) [29]. Gene expression was quantified with featureCounts (v2.0.6) [30].

### 2.7. Statistical Analysis

Differential expression analysis was performed by DESeq2 (v1.44.0) [31] in R (v4.4.1). A lfcThreshold of 1 was applied to identify genes with a significant change in expression. Shrinkage of log fold changes was performed using the “ashr” method to improve the interpretability of results [32]. To visualise the expression levels of genes across different groups, variance-stabilising transformation (vst) was utilised.

All visualisations were performed in R (v4.4.1) using RStudio (v2024.04.2 Build 764). Heatmaps were generated using the ‘pheatmap' package [33]. Volcano plots were created using the ‘EnhancedVolcano' package to highlight differentially expressed genes (DEGs) based on statistical significance and fold change [34]. Venn diagrams were constructed using ‘VennDiagram' package to illustrate the overlap and unique sets of DEGs across the various experimental conditions [35].

Pathway enrichment analysis was conducted using the ‘clusterProfiler' package (v4.12.6) [36] for Gene Ontology biological processes (GO/BPs) [37, 38] and KEGG pathways [39]. Cell marker over-representation analysis was performed using the CellMarker 2.0 database [40]. To investigate the involvement of DEGs in signalling pathways and to create protein–protein interaction (PPI) networks, we utilised the STRING-db (v12) online tool [41]. The ‘fgsea' package [42] was used for fast Gene Set Enrichment Analysis (GSEA) with Hallmark gene sets from the Molecular Signatures Database (MSigDB) [43–45].

## 3. Results

### 3.1. Secretome Analysis of SFV-Infected 4T1 Cells and Its Impact on Macrophage Transcriptome

**Figure 1:**
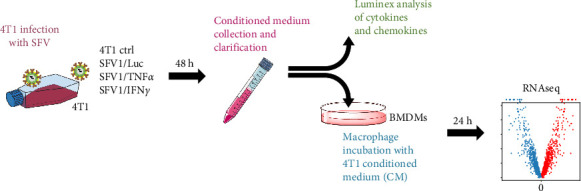
General concept of the study. 4T1 cells were infected with equal amounts of SFV/Luc, SFV/IFNγ and SFV/TNFα particles. After 48 h, the nontreated (ctrl) and SFV-infected 4T1 cell conditioned medium (CM) was collected and clarified. The cytokine and chemokine profiles of the CM were analysed by Luminex. 4T1 cell CM was then added to bone marrow-derived macrophages (BMDMs). After 24 h, BMDM transcriptome was analysed.

SFV infection induces significant alterations in the secretome of 4T1 murine mammary cancer cells, including changes in cytokine and chemokine profiles that can influence macrophage behaviour. To investigate these effects, we analysed the secretome of SFV-infected 4T1 cells and its subsequent impact on the transcriptomic landscape of macrophages. 4T1 cells were infected with SFV/Luc encoding firefly *Luc* gene (referred to as SFV or Luc) and two potential immunotherapy virus vectors, SFV/IFNγ and SFV/TNFα, to investigate the possibility of mitigating cancer cell effects on macrophages (Figure 1). The resulting SFV-infected 4T1 cell CM and uninfected 4T1 cell control were analysed to quantify the levels of 31 inflammatory cytokines and chemokines using the Luminex assay. BMDMs were incubated with 4T1 cell CM for 24 h, followed by transcriptome analysis of macrophages.

**Figure 2:**
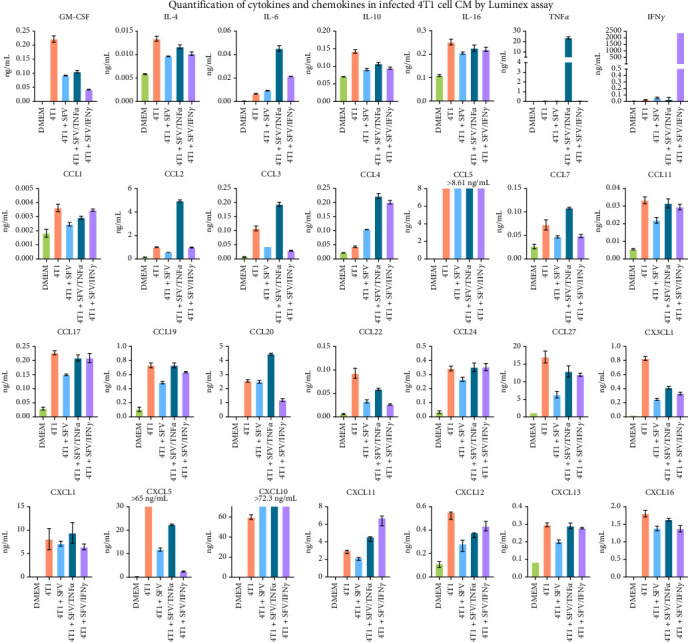
Cytokines and chemokines secreted by 4T1 cells infected with SFV/Luc (SFV), SFV/IFNγ and SFV/TNFα. The medium incubated with uninfected 4T1 cells (4T1) and the medium not exposed to cells (DMEM) were used as controls. Forty-eight hours postinfection, conditioned medium (CM) was collected, clarified and analysed for cytokine and chemokine profiles using Luminex. Data are presented as mean ± SD.

The uninfected 4T1 cell CM contained such cytokines as granulocyte-macrophage colony-stimulating factor (GM-CSF), IL-4, IL-6, IL-10 and IL-16 (*p*  < 0.001, Figure 2). 4T1 cell CM was also found to contain a set of chemokines, including CCL1, CCL2, CCL3, CCL5, CCL7, CCL11, CCL17, CCL19, CCL20, CCL22, CCL24, CCL27, CX3CL1, CXCL1, CXCL5, CXCL10, CXCL11, CXCL12, CXCL13 and CXCL16 (*p*  < 0.01, Figure 2). IL-1β, IL-2 and CCL12 were not found (Figure S1).

After 4T1 cell infection with SFV delivering inflammatory cytokine gene (SFV/IFNγ and SFV/TNFα), the 4T1 cells produced 2.4 µg/mL IFNγ and 23.6 ng/mL TNFα, respectively (Figure 2). 4T1 cell infection with SFV downregulated the amount of GM-CSF, IL-4, IL-10, IL-16, CCL1, CCL3, CCL11, CCL17, CCL19, CCL22, CCL27, CX3CL1, CXCL5 and CXCL12 quantified in 4T1 CM (*p*  < 0.05, Figure 2). Moreover, infection with SFV/IFNγ specifically inhibited GM-CSF, CCL20 and CXCL5 compared to the SFV group (*p*  < 0.0001). No proteins were found downregulated exclusively by SFV/TNFα.

4T1 cell infection with SFV upregulated the amount of CCL4 and CXCL10 (*p*  < 0.05). Moreover, infection with SFV/IFNγ and SFV/TNFα upregulated the amount of IL-6, CCL4 and CXCL11 (*p*  < 0.05, Figure 2). No proteins were found upregulated exclusively by SFV/IFNγ. On the other hand, infection with SFV/TNFα specifically upregulated the amount of IL-6, CCL2, CCL3, CCL7 and CCL20 (*p*  < 0.0001). The SFV did not significantly affect the natural expression of IFNγ, TNFα, IL-6, CCL20, CXCL1 and CXCL11 in 4T1 cells (*p*  > 0.05).

The resulting CM from uninfected, SFV/Luc-, SFV/IFNγ- and SFV/TNFα-infected 4T1 cells were then applied to BMDMs. This was followed by RNA sequencing to analyse gene expression changes and to identify affected pathways in macrophages. Expression profiles of 40,303 genes (by ENSEMBL IDs and 37,273 by gene names) were obtained, which, after the exclusion of low-expressed genes (10 counts in at least three samples), were reduced to 15,413 (15,153).

**Figure 3:**
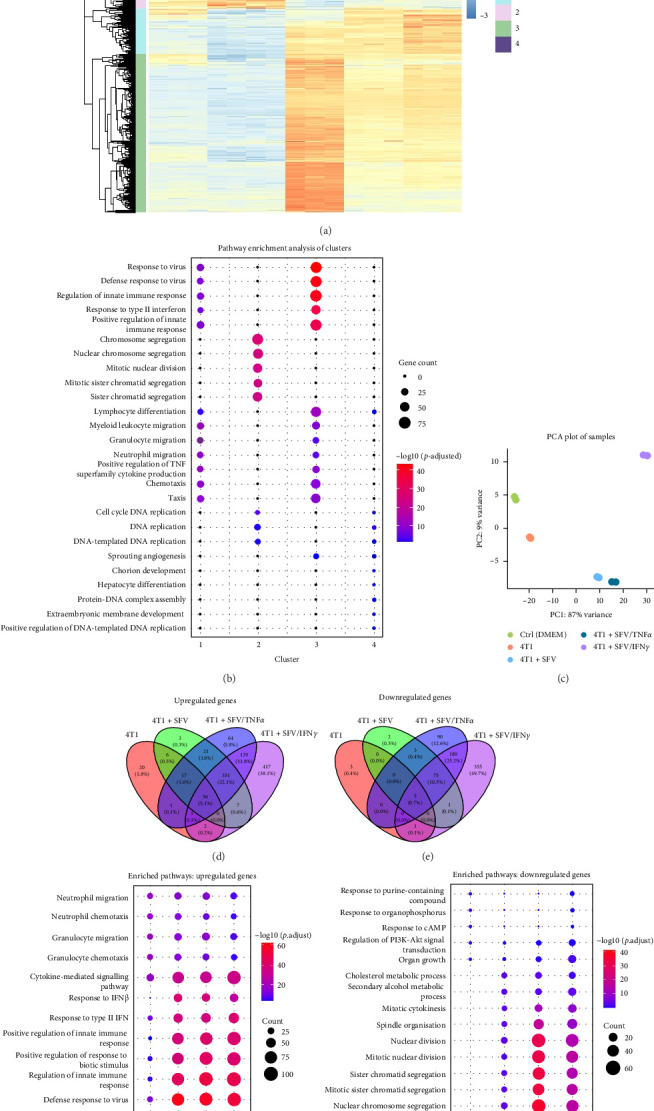
Gene expression analysis and hierarchical clustering of differentially expressed genes (DEGs) in macrophages treated with infected/uninfected 4T1 cell conditioned medium (CM). 4T1 cancer cells were infected with SFV/Luc (SFV), SFV/IFNγ or SFV/TNFα, and the resulting CM was collected and added to BMDMs. Uninfected 4T1 cell CM (4T1) and the medium not exposed to 4T1 cells (DMEM) were used as controls. BMDMs were cultured with respective CM for 24 h, followed by transcriptome analysis. (A) Heatmap of significant DEGs of macrophages (*p*  < 0.05, baseMean > 5, |log2FC| > 1.0). Cluster 1 and 3 genes are upregulated in all SFV groups, while Cluster 2 and 4 genes are downregulated in all SFV groups. (B) Gene Ontology (GO) biological process (BP) enrichment analysis of gene clusters derived from the heatmap. (C) Principal component analysis (PCA) of gene expression of the samples. (D) Venn diagram of upregulated genes (*p*  < 0.05, log2FC > 1.0). (E) Venn diagram of downregulated genes (*p*  < 0.05, log2FC < −1.0). (F) GO BP enrichment analysis of upregulated genes (*p*  < 0.05, log2FC > 1.0). (G) GO BP enrichment analysis of downregulated genes (*p*  < 0.05, log2FC < −1.0).

Principal component (PC) analysis of gene expression and hierarchical clustering of the significant DEGs revealed that gene expression profiles vary between groups (Figure 3A,C). Significant DEGs (*p*  < 0.05, baseMean > 5, |log2FC| > 1.0) were divided into four clusters, revealing that infection had a substantial impact on the macrophage expression profile (Figure 3A,B).

Genes upregulated in the SFV groups (Clusters 1 and 3) were associated with response to virus and immune cell migration, while genes downregulated in the SFV groups (Clusters 2 and 4) were associated with cell division processes. Additionally, delivering immunomodulating genes such as IFNγ or TNFα enhanced the effects of the infection. As a result, infection-upregulated genes were expressed at higher levels in the SFV/TNFα group (Cluster 1) and in the SFV/IFNγ group (Cluster 3).

The macrophage genes were then categorised as upregulated or downregulated and all groups were compared to the control (DMEM). In the 4T1 group, 103 upregulated and nine downregulated genes were identified. In the SFV/Luc group, 461 upregulated and 86 downregulated genes were identified. SFV/TNFα resulted in 640 upregulated and 353 downregulated genes, while SFV/IFNγ showed the largest number with 963 upregulated and 617 downregulated genes. Venn diagrams (Figure 3D,E) show that SFV/IFNγ has the most effect on gene expression as it has the largest number of uniquely up- and downregulated genes (38.1% and 49.7%, respectively). GO/BP analysis of the upregulated genes revealed that all groups show enrichment for neutrophil and granulocyte migration, with the 4T1 group displaying the highest significance level (p_granulocyte migration_ = 2.51 × 10^−19^) and SFV/IFNγ the lowest (*p*_granulocyte migration_ = 4.34 × 10^−8^; Figure 3F). In contrast, the infection groups showed enriched upregulation of genes related to the response to viruses, regulation of the immune response, cytokine-mediated pathways and responses to type I and II IFNs. Moreover, response to type II IFN is more significantly enriched in the SFV/IFNγ group (*p*_SFV/Luc_ = 6.39 × 10^−37^ vs. *p*_SFV/IFNγ_ = 7.84 × 10^−41^), as was expected.

GO/BP analysis of downregulated genes revealed the difference between the 4T1 and infection groups, with the effect increasing with the addition of immunomodulatory genes (Figure 3G). Thus, SFV infection downregulates genes associated with division and chromosome segregation. This effect is even more significant in the SFV/IFNγ and SFV/TNFα groups.

### 3.2. Impact of Uninfected 4T1 Cell Secretome on Macrophage Gene Expression

**Figure 4:**
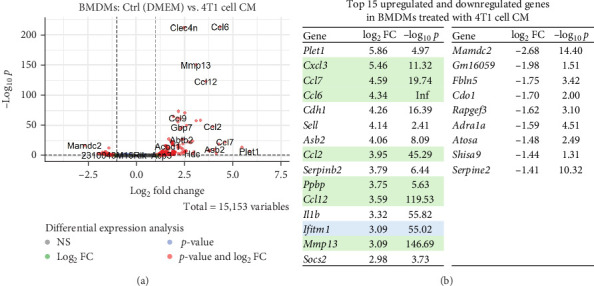
Alterations in the transcriptome of macrophages treated with 4T1 cell conditioned medium (CM). Differentially expressed genes in macrophages treated with 4T1 cell CM compared to untreated macrophages (DMEM). (A) Volcano plot. (B) The top of significantly upregulated and downregulated genes. Migration-associated genes are highlighted in green and IFN-upregulated genes are highlighted in blue. BMDMs, bone marrow-derived macrophages.

To evaluate the impact of the potential therapeutic vectors, we first conducted a detailed analysis of the genes upregulated by cancer cells (Figure 4A). Differential gene expression analysis between DMEM-treated macrophages (negative control) and macrophages exposed to 4T1 cell CM resulted in 112 significant DEGs (log2FC > ±1.0, *p*-adjusted < 0.05). There were significantly more upregulated (103 (92%), median log2FC = 1.89) than downregulated (9 (8%), median log2FC = −1.62) DEGs.

The top 15 upregulated genes included placenta-expressed transcript 1 (*Plet1*), matrix metallopeptidase 13 (*Mmp13*) and several chemokine genes such as *Cxcl3*, *Ccl2*, *Ccl6*, *Ccl7* and *Ccl12*, along with neutrophil chemoattractant *Ppbp* (Figure 4B). Other upregulated genes included the cell adhesion molecule *Asb2*, *Serpinb2*, *Il1b*, cadherin 1 gene *Cdh1* and selectin gene *Sell*, the IFN-upregulated antiviral protein *Ifitm1* and *Socs2*, which in macrophages suppresses inflammation by limiting the activation of the inflammasome signalling pathway [46].

Nine downregulated genes were identified, including MAM domain-containing 2 (*Mamdc2*), which is associated with increased susceptibility to Herpes Simplex virus 1 (HSV-1) infection and an impaired type I IFN-mediated antiviral response in Mamdc2-deficient mice [47]. Among other downregulated genes notable is *Fbln5*, whose expression correlates significantly with the infiltration of various immune cells into the tumour, including CD4+ T cells, M0 and M2 macrophages [48]. Other downregulated genes were ncRNA *Gm16059*, cysteine dioxygenase type I gene *Cdo1*, *Rapgef3*, adrenergic receptor alpha 1a gene *Adra1a*, *Atosa*, *Shisa9* and *Serpine2*.

### 3.3. Macrophage Gene Expression in Response to 4T1 Cell Infection With SFV/Luc

**Figure 5:**
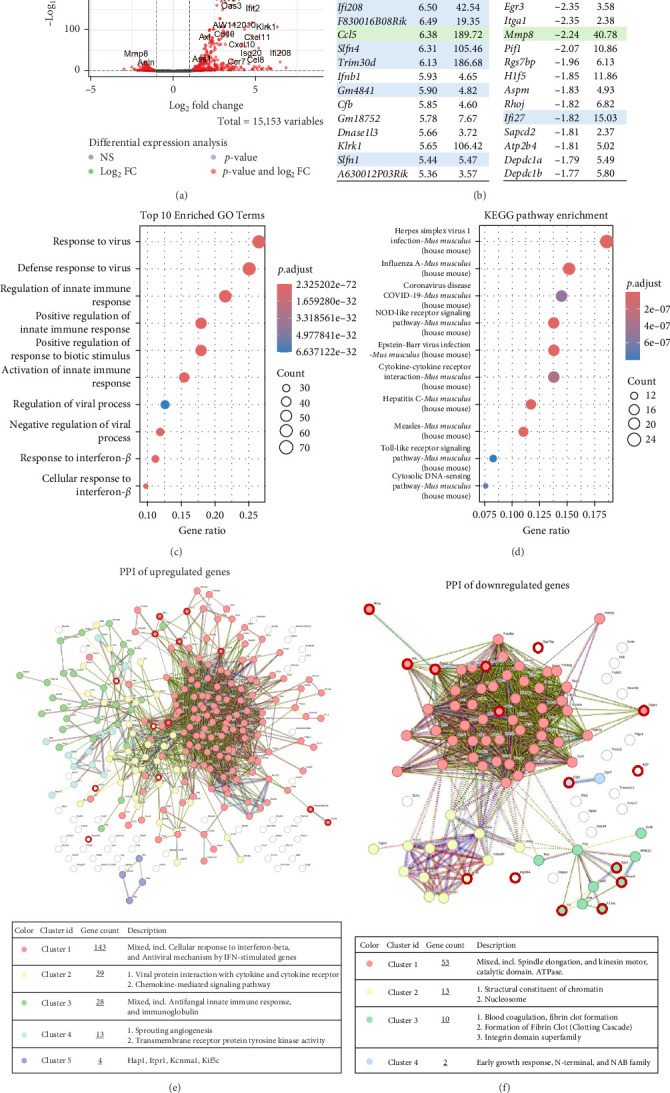
Macrophage gene expression in response to 4T1 cell infection with SFV/Luc virus. 4T1 cancer cells were infected with SFV/Luc (SFV) and the resulting conditioned medium (CM) was collected and added to bone marrow-derived macrophages (BMDMs). BMDMs were treated with infected/uninfected 4T1 cell CM for 24 h, followed by transcriptome analysis. (A) Differentially expressed genes in macrophages treated with 4T1 SFV/Luc-infected CM compared to CM from uninfected 4T1 cells. (B) The top 15 significantly upregulated and downregulated genes in the SFV/Luc group compared to the 4T1 group, with migration-associated genes highlighted in green and IFN-upregulated genes highlighted in blue. (C) The top 10 gene ontology (GO) biological process (BP) terms of upregulated genes with the highest statistical significance (*p*  < 0.05, log2FC > 1.0). (D) The top 10 enriched KEGG pathways of upregulated genes (*p*  < 0.05, log2FC > 1.0). (E) STRING protein–protein interaction (PPI) analysis of upregulated genes (*p*  < 0.05, log2FC > 1.0) divided into five clusters: IFNβ-regulated immune response (Cluster 1), viral protein interaction with cytokines and cytokine receptors (Cluster 2), innate immune response (Cluster 3), angiogenesis (Cluster 4) and unspecified Cluster 5, containing *Itpr1*, *Kcnma1*, *Hap1* and *Kif5c* genes. The red outlines highlight the top 15 upregulated genes. (F) STRING PPI analysis of downregulated genes (*p*  < 0.05, log2FC < −1.0) divided into four clusters: spindle elongation, chromatin and nucleosomes (Cluster 1 and 2), coagulation (Cluster 3) and early growth response (Cluster 4). The red outlines highlight the top 15 downregulated genes.

To investigate the effect of cancer cell infection with replication-deficient SFV on the macrophage transcriptome profile, a vector encoding the *Luc* gene was employed. Differential expression tests between the 4T1 and 4T1 + SFV/Luc groups resulted in 401 significant DEGs (log2FC > ± 1.0, *p*-adjusted < 0.05, Figure 5A). There were more upregulated (307 (77%), median log2FC = 2.31) than downregulated (94 (23%), median log2FC = −1.53) DEGs.

The top 15 upregulated genes included IFN-related genes like *Ifi213* and *Ifi208* and IFNγ-inducible GTPase genes *Gm4841* and *F830016B08Rik* (Figure 5B). Several classical IFN-stimulated genes, such as *Slfn1*, *Slfn4*, *Trim30d* and the chemokine gene *Ccl5*, were also upregulated. The upregulated genes included cytokine *Ifnb1*, complement factor B gene *Cfb*, killer cell lectin-like receptor gene *Klrk1* and ncRNA *A630012P03Rik*. Other notable upregulated genes were *Ms4a4c*, *Gm18752* and *Dnase1l3*.

The top 15 downregulated genes included *Mmp8*, coagulation factor genes *F3* and *F13a1*, transcription factor (TF) *Egr3*, integrin gene *Itga1* and helicase *Pif1*. Other notable downregulated genes were *Rgs7bp*, *Atp2b4* and *H1f5*, along with *Aspm* and *Sapcd2*, which are involved in mitotic spindle regulation and *Rhoj*, a GTPase linked to angiogenesis. Notably, IFN-upregulated gene *Ifi27*, which counterbalances innate immune responses to RNA virus infections, and DEP domain-containing protein genes *Depdc1a* and *Depdc1b* were downregulated.

GO/BP analysis showed that highly upregulated pathways are related to response to the virus and IFNβ (Figure 5C). KEGG pathway enrichment analysis (Figure 5D) revealed similarity of SFV/Luc infection with other known virus infections including ssRNA viruses Influenza A, SARS-CoV-2, Hepatitis C, Measles and dsDNA viruses—HSV1 and Epstein-Barr virus. Furthermore, Toll-like receptor (TLR) signalling, cytokine–cytokine receptor interaction, cytoplasmic NOD-like receptor signalling and cytosolic DNA sensing pathways were enriched.

To further explore the interactions among overlapping genes, we constructed a STRING network of physical protein associations. Upregulated gene (*p*  < 0.05, log2FC > 1.0) analysis identified 2695 interactions among 273 proteins (expected 267 interactions), yielding a PPI enrichment *p*-value of 10^−16^. Further gene division into clusters revealed SFV-upregulated IFNβ-regulated immune response (Figure 5E, Cluster 1), viral protein interaction with cytokines and cytokine receptors (Cluster 2), innate immune response (Cluster 3), angiogenesis (Cluster 4) and unspecified Cluster 5, containing *Itpr1*, *Kcnma1*, *Hap1* and *Kif5c* genes.

Downregulated gene analysis (*p*  < 0.05, log2FC < −1.0) identified 1167 interactions among 94 proteins (expected 108 interactions), yielding a PPI enrichment *p*-value of 10^−16^. The largest clusters were associated with spindle elongation, chromatin and nucleosomes (Figure 5F, Cluster 1 and 2), supporting SFV-mediated downregulation of cell division processes. Cluster 3 (10 genes) was associated with coagulation-related processes and Cluster 4 (2 genes) with early growth response. We can conclude that SFV infection of 4T1 cancer cells upregulates macrophage antiviral immune response.

### 3.4. Macrophage Gene Expression in Response to 4T1 Cell Infection With SFV/TNFα

**Figure 6:**
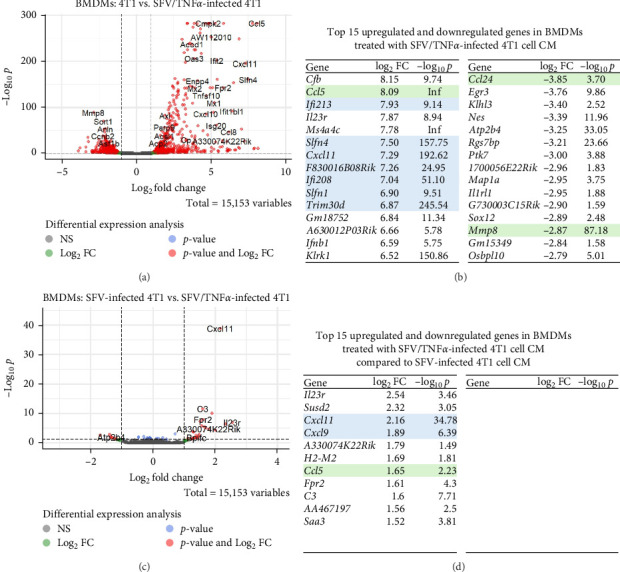
Macrophage gene expression in response to 4T1 cell infection with SFV/TNFα. (A) Differentially expressed genes (DEGs) in macrophages (BMDMs) treated with conditioned medium (CM) from 4T1 cells infected with SFV/TNFα, compared to CM from uninfected 4T1 cells. (B) The top 15 significantly upregulated and downregulated genes in the SFV/TNFα group compared to the 4T1 group, with migration-associated genes highlighted in green and IFN-upregulated genes highlighted in blue. (C) DEGs in macrophages (BMDMs) treated with CM from 4T1 cells infected with SFV/TNFα, compared to CM from 4T1 cells infected with SFV/Luc. (D) The top 15 significantly upregulated and downregulated genes in the SFV/TNFα group compared to the SFV/Luc group, with migration-associated genes highlighted in green and IFN-upregulated genes highlighted in blue.

SFV encoding TNFα and IFNγ are expected to inhibit the pro-tumorigenic effect of cancer cells and enhance macrophage anti-tumour gene expression, promoting an M1-like phenotype. Differential expression tests between the 4T1 and 4T1 + SFV/TNFα groups resulted in 826 significant DEGs in macrophages (Figure 6A, log2FC > ± 1.0, *p*-adjusted < 0.05). There were more upregulated (480 (58%), median log2FC = 2.23) than downregulated (346 (42%), median log2FC = −1.75) genes. The top 15 upregulated genes (Figure 6B) included IFN-upregulated genes *Ifi213* and *Ifi208*, *Slfn1*, *Slfn4*, *Trim30d*, *F830016B08Rik*, chemokine genes *Ccl5* and *Cxcl11*. The upregulated genes included cytokine *Ifnb1*, IL-23 receptor gene *Il23r*, complement factor B gene *Cfb* and killer cell lectin-like receptor gene Klrk1, along with the ncRNA *A630012P03Rik, Gm18752* and *Ms4a4c*. The top 15 downregulated genes included migration-associated genes *Ccl24* and *Mmp8*, TF genes *Egr3* and *Sox12*, IL-33 receptor *Il1rl1*. Other notable downregulated genes were *Rgs7bp*, *Atp2b4*, *Klhl3*, *Nes*, *Ptk7*, *1700056E22Rik*, *Map1a*, *G730003C15Rik*, *Gm15349* and *Osbpl10*.

However, the 4T1 + SFV/TNFα group exhibited minimal differences compared to the 4T1 + SFV/Luc group. Differential expression tests identified only 11 significant DEGs in the 4T1 + SFV/TNFα group *vs* the 4T1 + SFV/Luc group (Figure 6C, log2FC > ±1.0, *p*-adjusted < 0.05). All 11 genes were upregulated (11 (100%), median log2FC = 1.69). The upregulated genes (Figure 6D) included chemokine genes *Ccl5*, *Cxcl9* and *Cxcl11*, complement C3 gene, MHC I family gene *H2-M2*, as well as IL-23 and formyl peptide receptor genes *Il23r* and *Fpr2*. Other upregulated genes included *A330074K22Rik*, *AA467197*, *Saa3* and possible cytokine receptor *Susd2*.

### 3.5. Macrophage Gene Expression in Response to 4T1 Cell Infection With SFV/IFNγ

**Figure 7:**
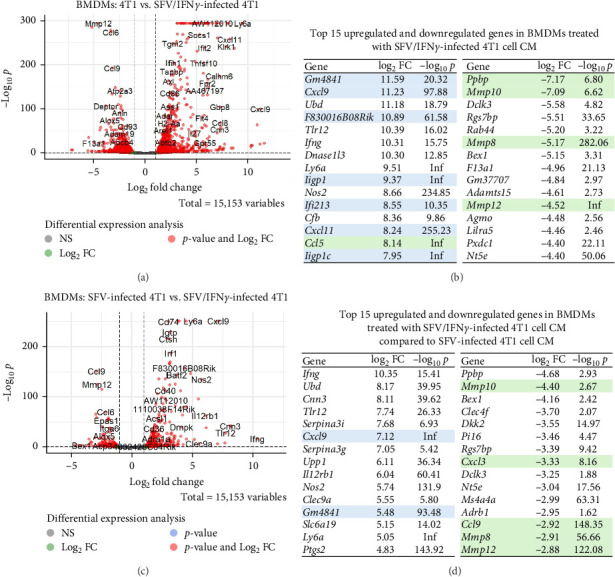
Macrophage gene expression in response to 4T1 cell infection with SFV/IFNγ virus. (A) Differentially expressed genes (DEGs) in macrophages (BMDMs) treated with conditioned medium (CM) from 4T1 cells infected with SFV/IFNγ, compared to CM from uninfected 4T1 cells. (B) The top 15 significantly upregulated and downregulated genes in the SFV/IFNγ group compared to the 4T1 group, with migration-associated genes highlighted in green and IFN-upregulated genes highlighted in blue. (C) DEGs in macrophages (BMDMs) treated with CM from 4T1 cells infected with SFV/IFNγ, compared to CM from 4T1 cells infected with SFV/Luc. (D) The top 15 significantly upregulated and downregulated genes in the SFV/IFNγ group compared to the SFV/Luc group, with migration-associated genes highlighted in green and IFN-upregulated genes highlighted in blue.

Differential expression tests between the 4T1 and 4T1 + SFV/IFNγ groups resulted in 1402 significant DEGs (Figure 7A, log2FC > ±1.0, *p*-adjusted < 0.05). There were more upregulated (814 (58%), median log2FC = 2.23) than downregulated (588 (42%), median log2FC = − 1.91) DEGs in the 4T1 + SFV/IFNγ group. The top 15 upregulated genes (Figure 7B) comprised *Ifng*, *Nos2*, TLR gene *Tlr12*, *Cfb* and Ubiquitin D gene *Ubd*. IFN-upregulated genes including *Iigp1*, *Ifi213*, *Iigp1c*, GTPase genes *Gm4841* and *F830016B08Rik*, chemokines *Ccl5*, *Cxcl9* and *Cxcl11*. Other notable upregulated genes included *Dnase1l3* and *Ly6a*. The top 15 downregulated genes included 4T1-upregulated neutrophil chemoattractant *Ppbp*, MMP genes—*Mmp8*, *Mmp10*, *Mmp12* and *Adamts15*, coagulation factor gene *F13a1*, member RAS oncogene family gene *Rab44*. Other notable downregulated genes were *Dclk3*, *Rgs7bp*, *Bex1*, *Gm37707*, *Agmo*, *Lilra5*, *Pxdc1* and *Nt5e*.

Conversely, differential expression tests between the 4T1 + SFV/Luc and 4T1 + SFV/IFNγ groups resulted in 396 significant DEGs (Figure 7C, log2FC > ±1.0, *p*-adjusted < 0.05). There were more upregulated (262 (66%), median log2FC = 2.08) than downregulated (94 (34%), median log2FC = −1.94) DEGs in the 4T1 + SFV/IFNγ group. The top 15 upregulated genes (Figure 7D) included *Ifng* and *Nos2*, IFN-inducible chemokine *Cxcl9*, IFNγ-inducible GTPase *Gm4841*, *Tlr12*, serine protease inhibitor genes *Serpina3i* and *Serpina3g*. Other upregulated genes comprised *Ubd*, *Cnn3*, *Upp1*, *Clec9a*, *Slc6a19*, *Ly6a* and *Ptgs2*, responsible for producing inflammatory prostaglandins and its upregulation is linked to increased cell adhesion, phenotypic changes, resistance to apoptosis and enhanced tumour angiogenesis [49]. The top 15 downregulated genes included chemokine genes *Cxcl3*, *Ccl9*, 4T1-upregulated neutrophil chemoattractant *Ppbp*, MMP genes—*Mmp8*, *Mmp10*, and *Mmp12*. Other downregulated genes included *Bex1*, *Clec4f*, *Dkk2*, *Pi16*, *Rgs7bp*, *Dclk3*, *Nt5e*, *Ms4a4a* and *Adrb1*.

### 3.6. Analysis of M1 and M2 Polarisation Markers

**Figure 8:**
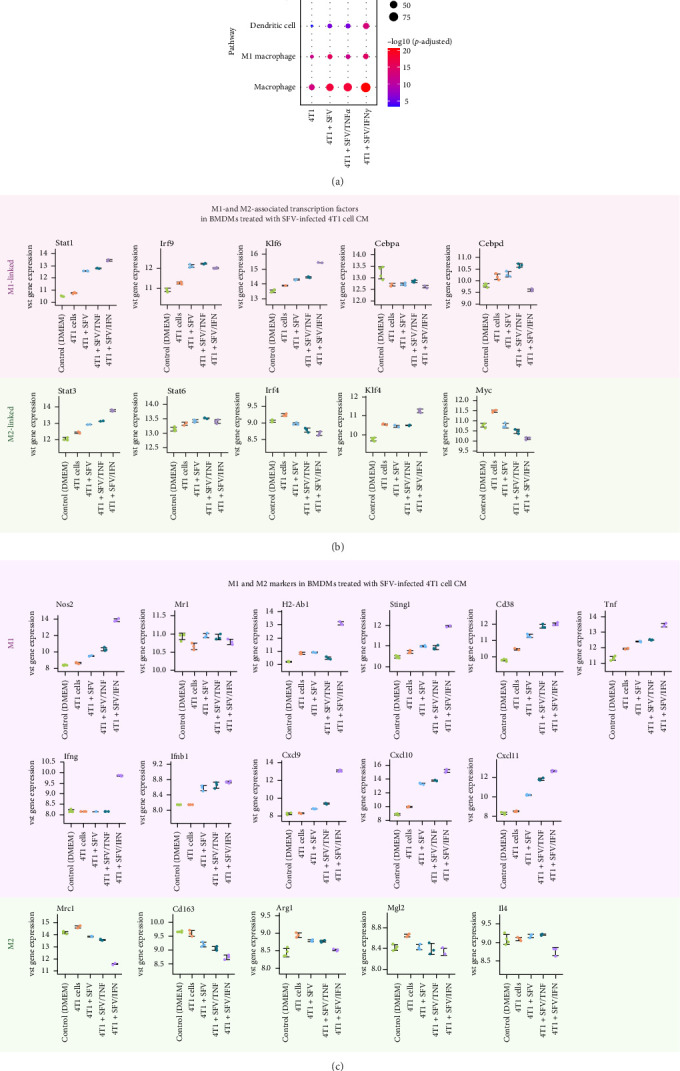
Analysis of macrophage polarisation through M1- and M2-associated gene expression. 4T1 cancer cells were infected with SFV, SFV/IFNγ and SFV/TNFα, the resulting conditioned medium (CM) was collected and added to BMDMs. BMDMs were treated with 4T1 cell CM for 24 h, followed by transcriptome analysis. (A) Cell marker over-representation analysis based on upregulated macrophage genes compared to control (untreated/DMEM BMDMs; *p*  < 0.05, log2FC > 1.0). (B) Expression of M1- and M2-associated transcription factor genes. (C) Expression of M1 and M2 marker genes. Gene expression levels transformed with variance-stabilising transformation (VST). Data are presented as mean ± SD.

Co-culture studies of macrophages with cancer cells demonstrate that cancer cells induce an M2-like immunosuppressive phenotype in macrophages [20]. To investigate the macrophage reprogramming potential, we analysed the enrichment of specific cell markers following treatment with SFV-infected 4T1 cell CM. The upregulated genes display significant enrichment of macrophage markers across all SFV groups (Figure 8A). Among them, the SFV/IFNγ group stands out with the strongest enrichment of M1 macrophage (*p*=1.4×10^−15^) and dendritic cell (*p*=7.4×10^−14^) markers. In contrast, the 4T1 group exhibits the highest significance for monocyte markers (*p*=6.2×10^−10^).

Analysis of M1- and M2-associated TFs revealed that 4T1 cell CM upregulates expression of such M2-linked TFs as *Klf4*, *Myc* and *Irf4* in macrophages, whereas SFV infection reduced *Myc* and *Irf4* (Figure 8B). Interestingly, *Klf4* was upregulated in SFV/IFNγ group compared to other groups. On the other hand, infection (the SFV group) significantly upregulated M1-linked TFs *Stat1*, *Irf9* and *Klf6* expression, as well as M2-linked *Stat3* expression. Moreover, *Stat1*, *Klf6* and *Stat3* were upregulated in the SFV/IFNγ group compared to the SFV/Luc (4T1 + SFV) group. Interestingly, M1-linked *Cebpa* expression was not affected by 4T1 cell CM or infection, while 4T1 cells upregulated M1-linked *Cepbd*. Infection with SFV/TNFα increased *Cepbd* levels, but infection with SFV/IFNγ reduced expression compared to the SFV/Luc (SFV) group. Thus, SFV therapy primarily activates TFs associated with the M1 macrophage phenotype; however, SFV/IFNγ also influences the expression of certain M2-associated TFs, indicating a multifaceted reprogramming of macrophages.

Furthermore, M1-associated gene expression was significantly upregulated by SFV infection: *Ifnb1*, *Cxcl10* and *Cxcl11* (Figure 8C). SFV/IFNγ upregulated expression of *Ifng*, *Nos2*, *H2-Ab1* (MHC II gene), *Sting1* and monokine induced by IFNγ (MIG) gene *Cxcl9*. Increased levels of CXCL9 and CXCL10 have been linked to enhanced infiltration of effector CD8+ T cells in various mouse and human cancers [50]. Some genes were slightly upregulated by 4T1 and even more upregulated after infection: *Tnf* and *Cd38*. *Mr1* (MHC I gene) expression was reduced by 4T1 treatment but returned to the nontreated level following infection.

Cancer cell CM upregulated expression of M2 markers *Mrc1* (CD206), *Arg1* and *Mgl2* in macrophages, but infection reduced it. Cd163 was not upregulated by 4T1 but was reduced by infection. However, only SFV/IFNγ infection could reduce the level of *Il4*. *Il10* and *Ido1* levels were very low (Figure S2), they did not meet the criteria for inclusion due to having low expression levels (fewer than 10 counts in at least three samples). M2-like phenotype-linked *Fizz1* (*Retnla*) and *Mgl1* (*Clec10a*) levels were consistently very low (Figure S2).

### 3.7. Analysis of SFV-Induced Changes in Pro-Tumorigenic Molecular Signatures of Macrophages

To gain deeper insights into the effects of SFV treatment on macrophage programming, we evaluated pro-tumorigenic TAM signatures based on previously published studies, specifically using the gene signatures identified by Hey et al. [51] and Cassetta et al. [52] (Figure S3A,B). As anticipated, the expression levels of genes such as *Mgat4a*, *Psd4*, *Clec4n*, *St3gal1*, *Nt5e* and *Dok2* were significantly elevated in the 4T1 group compared to the negative control (DMEM). Notably, SFV infection effectively downregulated the expression of these genes, indicating its potential to counteract pro-tumorigenic macrophage programming. However, several genes within these signatures are closely associated with inflammatory responses, which are expected to be upregulated during virus-mediated therapy. For instance, genes such as *Stat1*, *Tlr7*, *Cd40* and complement components *C1qa*, *C1qb* and *C1qc* were upregulated across all SFV infection groups.

**Figure 9:**
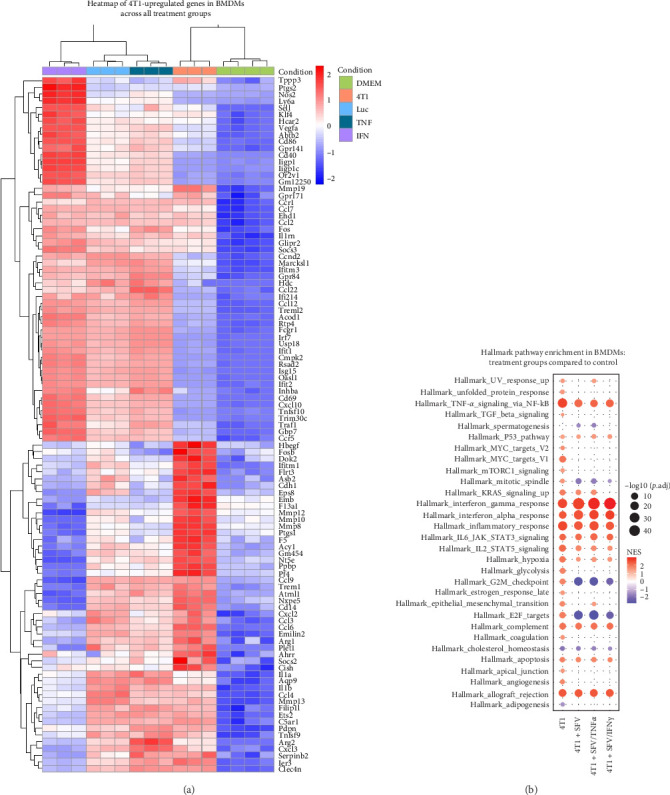
Comparison of pro/anti-tumorigenic molecular signatures of macrophages treated with SFV infected and uninfected 4T1 cell conditioned medium (CM). (A) Heatmap of 103 macrophage genes upregulated (*p*  < 0.05, log2FC > 1.0) following incubation with 4T1 cell CM and their comparison with SFV infected 4T1 cell CM: untreated (DMEM), 4T1 conditioned medium (4T1), SFV/Luc (Luc), SFV/TNFα (TNFα) and SFV/IFNγ (IFNγ). (B) MSigDB hallmarks of cancer-enriched transcripts in BMDMs in response to treatment with SFV-infected 4T1 cell CM (SFV – SFV/Luc, SFV/IFNγ and SFV/TNFα). Enrichment was calculated in comparison to untreated macrophages (DMEM).

To analyse the effectiveness of infection in mitigating 4T1-upregulated effects on macrophages, we selected macrophage genes upregulated (*p*  < 0.05, log2FC > 1.0) following incubation with 4T1 cell CM compared to untreated (DMEM) group (Figure 9A). The expression of these genes was examined across all groups. Of the 103 genes upregulated by 4T1, the SFV infection (the SFV/Luc group) reduced the expression of 46 genes (45%), while further increasing the expression of 57 genes (55%). The TNFα transgene expression (the SFV/TNFα group) reduced 43 genes and increased 60 genes. IFNγ transgene (SFV/IFNγ group) enhanced the effect of infection even more by reducing 51 genes and inducing 52 genes. SFV-downregulated genes are mostly associated with migration and invasion, including *Ccl3*, *Ccl6*, *Cxcl2*, *Mmp8*, *Mmp10*, *Mmp12*, *Mmp19* and *Ppbp. Ccl4*, *Ccl9* and *Mmp13* genes were downregulated exclusively by SFV/IFNγ. SFV-upregulated genes are mostly associated with immune response (*Cd40*, *Nos2*, *Ifit1*, *Irf7*, *Ifitm3*, *Ifi214*, *Ifit2*, *Oasl1*, *Trim30c* and *Cxcl10*).

TAMs help drive malignant tumour growth by enhancing epithelial cell invasion, migration and angiogenesis, which sustains tumour oxygen and nutrient supply. Thus, we explored the effect of the immunomodulating SFV vectors on each cancer-related marker. Several markers were reduced in SFV-treated groups, including Myc targets, angiogenesis, glycolysis, apical junction, coagulation, unfolded protein response and mTORC1 signalling (Figure 9B).

**Figure 10:**
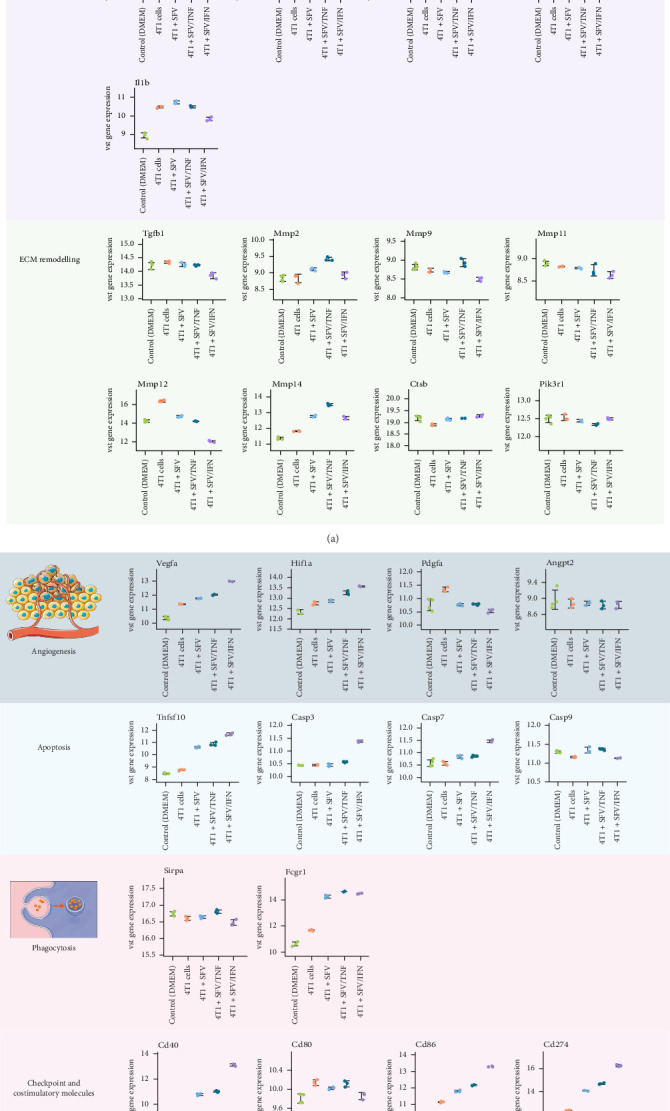
(A, B) Alterations of the macrophage gene expression associated with immunosuppression, tumour growth, angiogenesis, phagocytosis, apoptosis and expression of checkpoint molecules. 4T1 cancer cells were infected with SFV/Luc (SFV), SFV/IFNγ (SFV/IFN) and SFV/TNFα (SFV/TNF), the resulting conditioned medium (CM) was collected and added to BMDMs for 24 h, followed by transcriptome analysis. Control (DMEM): untreated BMDMs; 4T1 cells: BMDMs treated with 4T1 cell CM. VST, variance-stabilising transformation. Data are presented as mean ± SD.

Analysis of pro-tumorigenic markers (Figure 10) revealed that 4T1 upregulated expression of epithelial–mesenchymal transition (EMT)-related *Fn1* and *Timp1* in macrophages, but infection reduced it. The scavenger receptor gene *Stab1* was not upregulated by 4T1, but was decreased by infection. The *Il1rn* gene, strongly upregulated by 4T1 cancer cells, remained unaffected by infection. PGE2, known for inducing immunosuppression and promoting M2 macrophage polarisation [53, 54], showed no change in expression, with *Ptges2* unaffected by cancer cell medium or infection. In contrast, pro-tumorigenic *Ptgs2* expression was specifically upregulated by SFV/IFNγ. Furthermore, infection, particularly with SFV/TNFα, selectively upregulated the expression of *Marco—*a scavenger receptor gene, associated with the poor prognosis in many types of cancers.

TAM-derived IL-6 plays a crucial role in TAM-mediated cancer stem cell enrichment by activating STAT-3 signalling and influencing breast cancer cell migration and angiogenesis [55]; however, its expression was relatively low across all groups (Figure S2). Furthermore, the tumour-promoting TF *Runx3* previously shown to be upregulated in TAMs was downregulated by SFV/Luc and SFV/TNFα.

Analysis of tumour growth-related genes (Figure 10) revealed distinct effects of the cancer cell medium and viral infection on macrophage gene expression. The 4T1 cell-CM significantly upregulated the expression of *Fosl2* in macrophages, a change that was effectively reversed by viral infection. Similarly, the CM from 4T1 cells induced the expression of *Il1b*. However, this was specifically reduced by SFV/IFNγ treatment. In contrast, infection led to an upregulation of the pro-tumorigenic gene *Arid5a*, with the highest expression observed in the SFV/IFNγ group. Interestingly, while the levels of *Hgf* remained unchanged in the 4T1 group, they were reduced following infection, but subsequently upregulated by SFV/IFNγ. Notably, *Egf* levels were consistently very low across all conditions, indicating limited involvement in this experimental context.

Proteolytic enzymes that degrade the extracellular matrix (ECM) remodelling genes (Figure 10) were also assessed. SFV/TNFα affected the expression of MMPs the most by upregulating *Mmp2*, *Mmp9* and *Mmp14*. However, *Mmp11* was reduced following infection with SFV/Luc, SFV/IFNγ and SFV/TNFα. Other matrix metalloproteinases, including *Mmp1a*, *Mmp3* and *Mmp7*, were expressed at low levels and did not meet the criteria for inclusion in the analysis (Figure S2).

Given that TAMs are critical drivers of (lymph)angiogenesis, we examined the impact of infection on key angiogenic factors (Figure 10). In the 4T1 group, *Pdgfa*, *Vegfa* and *Hif1a* expression was upregulated. SFV infection significantly reduced 4T1-upregulated *Pdgfa* levels, while increasing *Vegfa* and *Hif1a* expression, markers commonly associated with hypoxia and tumour progression. However, *Angpt2* levels were unaffected and *Tie2* receptor expression remained extremely low across all conditions. These findings suggest that while infection inhibits certain pro-angiogenic factors, others are upregulated by SFV cancer cell infection and further stimulated with the presence of TNFα and IFNγ.

We have further analysed the expression of markers linked to phagocytosis and apoptosis. The Fc receptor gene *Fcgr1* was slightly upregulated by 4T1 and even more upregulated after infection. *Tnfsf10* (TRAIL gene), *Casp3* and *Casp7* were significantly upregulated by infection. *Sirpa* and *Casp9* expression did not change significantly after 4T1 medium and infection.

Furthermore, we investigated the effects of SFV infection on various immune checkpoint genes and co-stimulatory molecules in macrophages (Figure 10). Notably, SFV infection upregulated *Cd274* (PD-L1), but did not upregulate expression of *Pdcd1* (PD-1) and *Pdcd1lg2* (PD-L2; Figure S2). *Cd274* expression was increased by SFV/IFNγ even more. In contrast, the infection led to a reduction in the expression of the *Vsir* gene, which encodes the V-domain Ig suppressor of T cell activation (VISTA) that functions as a negative immune checkpoint regulator, contributing to macrophage-mediated immune suppression. In the case of co-stimulatory molecules, *Cd40* and *Cd86* were both upregulated by infection, with IFNγ further amplifying this effect. The activation of these molecules has been associated with a shift in macrophage phenotype from an immunosuppressive to a pro-inflammatory state, thereby enhancing anti-tumour immunity [56]. Importantly, expression of *Cd80* was not significantly affected by either cancer cell medium or infection, and B7-H4 (*Vtcn1*) was very low in any of the experimental groups (Figure S2). Overall, we can conclude that while inducing anti-tumour-associated *Cd40* and *Cd80* and reducing pro-tumorigenic VISTA, SFV also upregulated PD-L1 which could interfere with the therapeutic effect.

**Figure 11:**
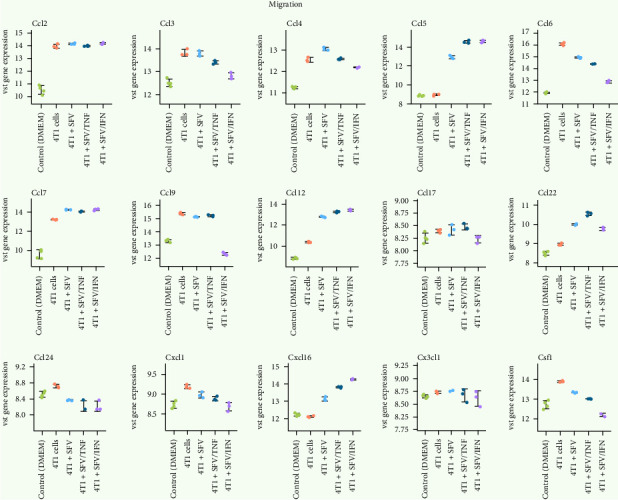
Alterations of the macrophage gene expression associated with migration and invasion. 4T1 cancer cells were infected with SFV/Luc (SFV), SFV/IFNγ and SFV/TNFα, the resulting conditioned medium (CM) was collected and added to BMDMs for 24 h, followed by transcriptome analysis. Control (DMEM): untreated BMDMs; 4T1 cells: BMDMs treated with 4T1 cell CM. VST, variance-stabilising transformation. Data are presented as mean ± SD.

Eliminating invasion, metastasis and EMT is essential for effective cancer therapies. Interestingly, 4T1 upregulated the expression of all MHC II^low^ M2-like TAM-associated [57] monocyte-recruiting chemokines, including gene expression of CCR2 ligands *Ccl2* and *Ccl12*, CCR1 ligands *Ccl6* and *Ccl9*, CCR1/2/3 ligand *Ccl7*, CCR5 ligand *Ccl4* and CCR1/CCR5 ligand *Ccl3* (Figure 11). The *Ccl2* gene, strongly upregulated by 4T1 cancer cells, remained unaffected by infection. However, infection reduced the expression of several chemokines upregulated by 4T1, including monocyte recruitment factors *Csf1*, *Ccl3*, *Ccl6*, *Ccl9*, *Ccl24* and EMT-associated *Cxcl1* [58]. Interestingly, metastasis-related *Ccl9* was upregulated by 4T1 and downregulated exclusively by SFV/IFNγ. *Ccl4* was upregulated by SFV/Luc, but reduced with SFV/IFNγ infection. In contrast, 4T1-upregulated *Ccl7*, *Ccl12* and *Ccl22* (most strongly upregulated by SFV/TNFα) were even more upregulated after infection. Furthermore, infection led to an increase in the expression of cancer progression-related chemokines *Ccl5* and *Cxcl16*, being upregulated most prominently by SFV/TNFα and SFV/IFNγ. *Cxcl15* (IL-8), *Ccl20*, *Csf2* (GM-CSF), *Csf3*, *Il34*, *Sox9* and *Hc* (C5a gene) were considered to be low expressed (Figure S2).

## 4. Discussion

In this study, we have investigated the effects of SFV immunotherapy vectors on the macrophage transcriptome. By applying CM from SFV-infected cancer cells to BMDMs, we characterised alterations in macrophage gene expression and identified significant pathways impacted by the SFV-based therapeutic approach. Our findings demonstrate that SFV vectors, particularly those engineered to deliver TNFα or IFNγ, can reprogramme macrophages towards an anti-tumour phenotype, with substantial implications for cancer immunotherapy. On the other hand, our findings clearly highlight the complex interplay between cancer cell-derived factors and SFV-mediated immunotherapy vectors in regulating genes associated with tumour progression, underlining the dual role of immune activation in the TME.

The data indicate that 4T1 cancer cells strongly influence macrophage behaviour through the cancer cell secretome, promoting a pro-tumorigenic phenotype characterised by enhanced chemotaxis, migration and angiogenesis-associated gene expression. Previous studies reported that 4T1 cell CM upregulates the expression of migration and chemotaxis genes [5, 59]. For instance, in breast cancer, 4T1 cells secrete GM-CSF, stimulating macrophages to produce CCL2, which recruits monocytes and promotes metastasis [8, 9]. Moreover, CM from B16 melanoma cells induces M2-like polarisation in BMDMs through IL-10 and TGFβ [60]. Similarly, in non-small cell lung cancer (NSCLC), IL-37 secreted by cancer cells drives this polarisation [3]. Additionally, tumour-modified metabolism, such as the Warburg effect, fosters the M2-like phenotype by altering macrophage activity, driven by metabolites like lactic acid, which enhance immunosuppression [61].

SFV infection markedly altered the secretory profile of 4T1 cells, reducing the levels of several immunosuppressive cytokines and chemokines, such as IL-4 and IL-10, while inducing inflammatory mediators like CCL4 and CXCL10 (Figure 2). These changes likely underpin the observed reprogramming of macrophages towards a more inflammatory and anti-tumorigenic state. Furthermore, SFV infection of cancer cells downregulated several migration- and invasion-associated genes in macrophages (e.g., *Mmp8*, *Mmp10* and *Mmp12*), particularly in the SFV/IFNγ group, suggesting that this approach may inhibit cancer cell dissemination and metastasis. Additionally, hallmark pathways associated with tumour progression, including angiogenesis, glycolysis, apical junctions, coagulation, unfolded protein response, Myc signalling and mTORC1 signalling [62] were significantly suppressed by SFV treatment. This broad reduction in cancer-promoting pathways suggests that SFV treatment may suppress key mechanisms driving tumour progression and malignancy.

Comparing these findings with other viral-based cancer immunotherapies, adenovirus-based vectors have also shown the ability to remodel the TME. For example, oncolytic adenoviruses armed with GM-CSF have demonstrated the ability to recruit and activate dendritic cells, thereby facilitating robust anti-tumour immunity [63–65]. However, the expression of GM-CSF in some contexts has been associated with pro-tumorigenic roles, indicating the complexity of cytokine interactions within the TME [66, 67]. Notably, SFV vectors, particularly when combined with IFNγ, appear to suppress GM-CSF and other broad-spectrum tumour-related pathways while inducing a pro-inflammatory state in macrophages, highlighting the complexity and multifaceted nature of TAM regulation. Similarly, vaccinia virus-based therapies have demonstrated potent immunostimulatory effects, including the upregulation of inflammatory cytokines and enhanced antigen presentation [68]. However, vaccinia viruses often induce systemic inflammation, highlighting a potential advantage of replication-deficient vectors, which localise their effects more effectively within the TME. Thus, while several oncolytic vectors were shown to be able to reprogramme TME and suppress tumour-promoting pathways, the potential of replication-deficient vectors is less investigated. SFV vectors exhibit a balance of immunostimulatory potency and safety, making them a promising candidate for further development in cancer immunotherapy.

Macrophage plasticity plays a key role in shaping the TME, where TAMs adopt an M2-like pro-tumorigenic phenotype. Our study confirms that 4T1-conditioned media induce the expression of M2 markers such as *Arg1*, *Mrc1* (CD206) and *Ccl22*. Interestingly, similar to previous studies in a glioblastoma mouse model [69], we have found the upregulation of *Cdh1* (Figure 4B), *Arg1*, *Mrc1* (Figure 8C) and *F13a1*, *Hmox1* and *Il1r2* (Figure S2) gene expression in macrophages after incubation with 4T1 cell CM. This suggests that TAM cancer-promoting mechanisms may be conserved across different tumour types. However, SFV treatment, especially with the IFNγ transgene, effectively counteracted this polarisation, inducing M1 markers like *Nos2*, *Cxcl9*, *Cxcl10* and *Cxcl11*. This reprogramming could enhance anti-tumour immune responses by promoting the recruitment and activation of effector T cells within the tumour milieu.

Notably, macrophages treated with SFV/TNFα- and SFV/IFNγ-infected 4T1 cell CM exhibited distinct transcriptional profiles. While SFV/TNFα treatment appeared to promote ECM remodelling and chemokine expression (Figure 10), SFV/IFNγ treatment enhanced the expression of interferon-stimulated genes (ISGs), inflammatory cytokines and antigen-presentation-related genes (Figure 8). These results align with previous studies indicating that IFNγ induces a robust immune response capable of enhancing tumour-associated antigen presentation and activating CTLs [70]. Therefore, treatment with SFV vectors could impact the cytokine and chemokine signalling profile in the TME, potentially altering macrophage behaviour and immune responses.

When considering SFV vector therapeutic expectations, SFV/TNFα and SFV/IFNγ serve different functions in cancer treatment. SFV/TNFα is particularly advantageous when tissue remodelling is required, as it enhances the expression of chemokines and MMPs, facilitating ECM degradation and subsequent tissue restructuring. However, even though SFV/TNFα-treated cancer cells secreted TNFα, IL-6, CCL2, CCL3, CCL4, CCL7 and CCL20, macrophages treated with this medium were similar to the SFV/Luc group and differed only in the induction of *Mmp2*, *Mmp9*, *Mmp14*, *Ccl22*, *Cepbd* and *Marco*. Despite being well-documented in previous studies, the effects of TNFα on macrophages—including the induction of *Il1b* and *Plaur* [71]—were not observed in this study.

In contrast, SFV/IFNγ is more effective in promoting inflammatory responses and inhibiting cell migration, making it suitable for regulating immune responses and limiting tumour metastasis. In our previous study, SFV/IFNγ therapy showed promising results in vivo, significantly decreasing the 4T1 tumour size in mice and enhancing T-cell infiltration into the tumour [17]. In 4T1 tumours treated with SFV/IFNγ and TLR2/1 ligand Pam3SCK4, the amount of CTLs was increased and the amount of Tregs was significantly reduced [17]. Moreover, treatment with SFV/Luc also slightly decreased the tumour size and increased CTL infiltration. This study confirms that SFV increases the expression of T-cell chemoattractant genes *Cxcl9*, *Cxcl10*, *Cxcl11* and immune checkpoint molecule gene *Cd40*, promoting anti-tumour immunity. IFNγ has been shown to induce these genes [72]. Additionally, the SFV/IFNγ group exhibited the lowest significance for neutrophil and granulocyte migration which was induced by cancer cells and may indicate the inhibition of cancer cell-induced processes (Figure 3F). Hence, we conclude that the IFNγ transgene synergises with the SFV vector, thereby upregulating T-cell response-inducing genes. On top of that, SFV/IFNγ treatment upregulates the expression of macrophage MHC II genes (*H2-Ab1*) and therefore can activate an adaptive immune response against cancer.

On the other hand, although the SFV treatment primarily activates TFs associated with the M1 macrophage phenotype (Figure 8B); it also influences the expression of certain M2-associated TFs, suggesting a multifaced reprogramming of macrophages. This dual modulation may reflect a balanced immune activation, with SFV/IFNγ showing the strongest potential to skew macrophages toward a pro-inflammatory and anti-tumorigenic state, while mitigating immunosuppressive influences within the TME. Furthermore, the use of pro-tumorigenic TAM signatures based on previously published studies (Figure S3) [51, 52] is not optimal in the context of virus-related immunotherapy. While SFV treatment appears to mitigate certain tumour-promoting effects, the pro-tumorigenic TAM score becomes less reliable for assessing virus-treated samples, as it does not account for the beneficial aspects of inflammatory activation associated with vector immunotherapy. This underscores the need for refined evaluation metrics that can distinguish between pro-tumorigenic inflammation and therapeutic immune activation within the TME.

The analysis of checkpoint molecules revealed that SFV with and without cytokine transgenes upregulated PD-L1 gene (*Cd274*) expression in macrophages with the highest expression after the SFV/IFNγ treatment. It is known that type I and type II IFNs induce PD-L1 expression [73]. Furthermore, several studies showed that IFNγ induces macrophage *Cd274* expression [74–76]. While PD-L1 expression is often associated with immune suppression, it also reflects activation of the interferon pathway, a double-edged sword in cancer immunotherapy with IFNs. Combining SFV/IFNγ with anti-PD-L1 or other immune checkpoint inhibitors may, therefore, enhance therapeutic efficacy by simultaneously activating CTLs and mitigating suppressive signals within the TME. Previous studies have demonstrated that PI3K inhibition amplifies IFNγ-mediated anti-tumour responses by downregulating PD-L1 [77], suggesting a potential combinatorial strategy for future investigations with SFV/IFNγ and anti-PD-L1. Furthermore, multiple studies have demonstrated an enhanced efficacy of virus-based therapy when combined with checkpoint inhibitors [78–81].

Our findings highlight the complexity of macrophage polarisation and underscore the need for further studies to optimise SFV-based therapies for precise immune modulation in the context of chronic inflammation and immune tolerance of the TME. Evaluating macrophage phenotypes and distinguishing between pro- or anti-tumour immune responses is complex, as many inflammatory genes are categorised as pro-tumorigenic under chronic tumour-associated inflammation [82]. The TME is characterised by sustained inflammation, which not only fails to elicit an effective anti-tumour immune response but also actively facilitates tumour progression. For example, SFV infection significantly reduced 4T1-upregulated *Pdgfa* levels, while inducing *Vegfa* and *Hif1a* expression (Figure 10), markers commonly associated with hypoxia and tumour progression. Therefore, more studies are necessary to clarify the signature genes of pro- and anti-tumour phenotypes.

Our previous studies demonstrated that macrophage co-cultivation with 4T1 cancer cell spheroids leads to the upregulation of CD38 and IFNβ in macrophages [20]. In the current study, we observed similar upregulation of *Cd38*, while *Ifnb1* did not show increased expression. CD38 exhibits dual effects in cancer biology. On one hand, CD38 expression is associated with the recruitment of immunosuppressive cells, including Tregs and MDSCs, and studies indicate that CD38 facilitates cancer cell migration, proliferation and colony formation [83–86]. Conversely, high densities of CD38+ macrophages have been linked to improved prognosis in certain cancers [87]. Furthermore, CD38 has been identified as a reliable marker for M1 macrophages under reprogramming conditions [20, 88]. In agreement with these findings, *Cd38* was upregulated after macrophage incubation with 4T1 CM. Moreover, 4T1 treatment with SFV vectors enhanced the expression even more, with the highest *Cd38* levels in the SFV/IFNγ group (Figure 8C). It remains unclear whether *Cd38* should be inhibited or stimulated for therapeutic purposes.

We previously showed the downregulation of MHC II in macrophages after co-cultivation with 4T1 spheroids [20], which was not observed in this study. Additionally, while earlier findings reported downregulation of NO, CCL22, CXCL12 and CXCL16, these genes were not downregulated with *Ccl22* being upregulated in the current analysis (log2FC = 2.39, *p*=1.59 × 10^−9^). The absence of respective gene expression inhibition could be linked to indirect contact with cancer cells (the use of CM) in contrast to co-cultures BMDMs with cancer cells in previous studies. Importantly, 4T1 cells express receptors for CCL17 and CCL22 (CCR4) [89]. Consequently, the observed reduction in CXCL12 and CCL22 levels in the presence of cancer cell spheroids may be partially due to the binding of these chemokines to their corresponding receptors.

Based on our findings, several hypotheses for further vector-related therapy development can be proposed. (i) SFV/IFNγ-induced macrophage reprogramming may synergise with other TME-targeted therapies, such as TLR agonists or CTL-activating agents (checkpoint inhibitors), to achieve durable anti-tumour responses. (ii) The conserved upregulation of TAM-promoting genes (e.g., *Arg1*, *Mrc1* and *F13a1*) across tumour types suggests that SFV-based therapies could have broader applicability in reprogramming TAMs in other malignancies, such as glioblastoma or lung cancer. (iii) While TNFα promotes ECM remodelling, potentially aiding therapeutic delivery, its role in enhancing metastasis-associated factors (e.g., MMP2 and MMP9) warrants careful optimisation of cytokine combinations to maximise therapeutic benefit. (iv) To our knowledge, this is the first study evaluating an indirect (through infected cell CM) effects on macrophage transcriptome simulating the simplified TME conditions under virus-based therapy, which conceptually can be applied to other viral vectors and other TME components (e.g., T cells and NK cells), broadening the impact of this study.

In conclusion, our results underscore the potential of SFV-based immunotherapy vectors to modulate the TME by reprogramming macrophages towards an anti-tumour phenotype. SFV/IFNγ demonstrated a particularly robust ability to induce inflammatory pathways and suppress pro-tumorigenic signals, making it a promising candidate for further development in combination therapies. These findings lay the groundwork for exploring SFV vectors in preclinical models of metastatic and immunosuppressive cancers, advancing the field of virus-based cancer immunotherapy.

## Data Availability

The raw sequencing data generated in this study are available at the European Nucleotide Archive (ENA) under the study accession number PRJEB85327. All data generated or analysed during this study are included in this article. Further inquiries can be directed to the corresponding author.

## Supporting Information

Supporting Information Figure S1. IL-1β, IL2 and CCL12 secretion by 4T1 cells infected with SFV/Luc (SFV), SFV/IFNγ and SFV/TNFα. The medium incubated with 4T1 cells (4T1) and the medium not exposed to cells (DMEM) were used as controls. After 48 h, conditioned medium (CM) was collected, clarified and analysed for cytokine and chemokine profiles using Luminex. Data are presented as mean ± SD. Figure S2. Variance-stabilising transformation (vst) for expression of macrophage genes that did not meet the criteria for inclusion due to having low expression levels (fewer than 10 counts in at least three samples). 4T1 cancer cells were infected with SFV, SFV/IFNγ and SFV/TNFα, the resulting conditioned medium (CM) was collected and added to BMDMs for 24 h, followed by transcriptome analysis. Data are presented as mean ± SD. Figure S3. Expression of pro-tumourigenic TAM signatures in BMDMs treated with conditioned medium (CM) from 4T1 cells infected with SFV/Luc (Luc), SFV/TNFα (TNF) and SFV/IFNγ (IFN). 4T1 group represents BMDMs incubated with uninfected cell CM, DMEM—untreated BMDMs. BMDMs were treated with 4T1 cell CM for 24 h, followed by transcriptome analysis. (A) Heatmap of selected pro-tumorigenic TAM genes defined by Hey et al. [51] and (B) heatmap of selected protumorigenic TAM genes defined by Cassetta et al. [52].
