# Supplementary material for: Macrophage Transcriptomic Alterations Driven by Alphavirus-Based Cancer Immunotherapy Vectors
Source: J Immunol Res. 2025 Jun 13;2025:6573891. doi: 10.1155/jimr/6573891 (PMC12181664; doi:10.1155/jimr/6573891)
Supplement: Supporting Information — Figure S1. IL-1β, IL2 and CCL12 secretion by 4T1 cells infected with SFV/Luc (SFV), SFV/IFNγ and SFV/TNFα. The medium incubated with 4T1 cells (4T1) and the medium not exposed to cells (DMEM) were used as controls. After 48 h, conditioned medium (CM) was collected, clarified and analysed for cytokine and chemokine profiles using Luminex. Data are presented as mean ± SD. Figure S2. Variance-stabilising transformation (vst) for expression of macrophage genes that did not meet the criteria for inclusion due to having low expression levels (fewer than 10 counts in at least three samples). 4T1 cancer cells were infected with SFV, SFV/IFNγ and SFV/TNFα, the resulting conditioned medium (CM) was collected and added to BMDMs for 24 h, followed by transcriptome analysis. Data are presented as mean ± SD. Figure S3. Expression of pro-tumourigenic TAM signatures in BMDMs treated with conditioned medium (CM) from 4T1 cells infected with SFV/Luc (Luc), SFV/TNFα (TNF) and SFV/IFNγ (IFN). 4T1 group represents BMDMs incubated with uninfected cell CM, DMEM—untreated BMDMs. BMDMs were treated with 4T1 cell CM for 24 h, followed by transcriptome analysis. (A) Heatmap of selected pro-tumorigenic TAM genes defined by Hey et al. [51] and (B) heatmap of selected protumorigenic TAM genes defined by Cassetta et al. [52]. [file 6573891.f1.docx]

***Supplementary Material for Research Article***

**Macrophage Transcriptomic Alterations Driven by Alphavirus-Based Cancer Immunotherapy Vectors**

Ksenija Korotkaja^a^, Dārija Lapiņa^a^, Zhanna Rudevica^a^, Anna Zajakina^a^

^a^ Cancer Gene Therapy Group, Latvian Biomedical Research and Study Centre, Ratsupites Str. 1 k. 1, Riga, Latvia, LV-1067

Corresponding Author: Anna Zajakina

E-mail address: anna.zajakina@gmail.com

Keywords: bone marrow-derived macrophages, Semliki Forest virus, alphavirus, tumour microenvironment, cytokine gene delivery

**Figure S1.** IL-1β, IL2 and CCL12 secretion by 4T1 cells infected with SFV/Luc (SFV), SFV/IFNγ, and SFV/TNFα. The medium incubated with 4T1 cells (4T1) and the medium not exposed to cells (DMEM) were used as controls. After 48h, conditioned medium (CM) was collected, clarified and analysed for cytokine and chemokine profiles using Luminex. Data are presented as Mean ± SD.

**
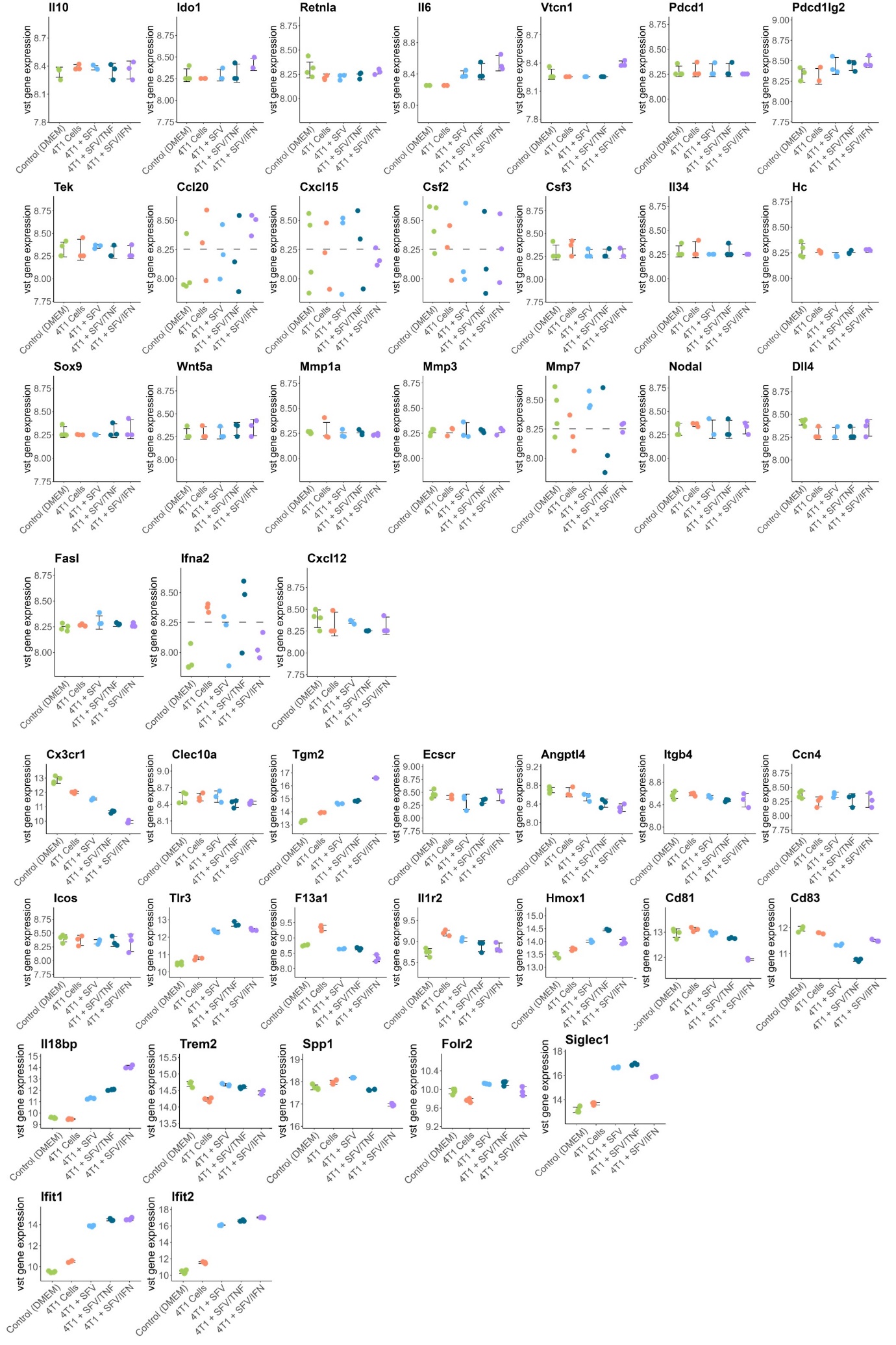
**

**Figure S2.** Variance-stabilising transformation (vst) for expression of macrophage genes that did not meet the criteria for inclusion due to having low expression levels (fewer than ten counts in at least three samples). 4T1 cancer cells were infected with SFV, SFV/IFNγ, and SFV/TNFα, the resulting conditioned medium (CM) was collected and added to BMDMs for 24h, followed by transcriptome analysis. Data are presented as Mean ± SD.


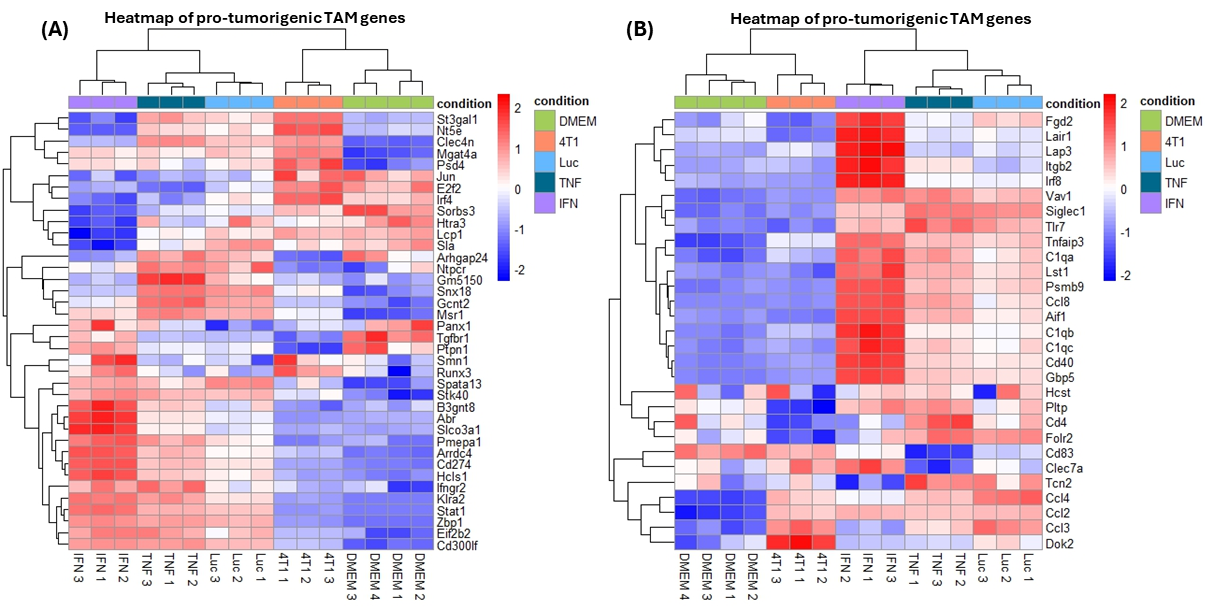


**Figure S3.** Expression of pro-tumourigenic TAM signatures in BMDMs treated with supernatants from 4T1 cells infected with SFV/Luc (Luc), SFV/TNFα (TNF), and SFV/IFNγ (IFN). 4T1 group represents BMDMs incubated with uninfected cell supernatant, DMEM – untreated BMDMs. BMDMs were treated with 4T1 cell supernatants for 24 h, followed by transcriptome analysis. **(A)** Heatmap of selected pro-tumorigenic TAM genes defined by Hey et al. [51]; **(B)** Heatmap of selected pro-tumorigenic TAM genes defined by Cassetta et al. [52].
